# Antimicrobial growth promoters approved in food-producing animals in South Africa induce shiga toxin-converting bacteriophages from *Escherichia coli* O157:H7.

**DOI:** 10.1186/s13099-023-00590-9

**Published:** 2023-12-06

**Authors:** Nomonde F. N. Ngoma, Mogaugedi N. Malahlela, Munyaradzi C. Marufu, Beniamino T. Cenci-Goga, Luca Grispoldi, Eric Etter, Alan Kalake, Musafiri Karama

**Affiliations:** 1 Department of Paraclinical Sciences, Faculty of Veterinary Science, Veterinary Public Health Section, University of Pretoria, Onderstepoort, 0110 South Africa; 2 Department of Veterinary Tropical Diseases, Faculty of Veterinary Science, University of Pretoria, Pretoria, South Africa; 3 Departimento di Medicina Veterinaria, Laboratorio di Ispezione Degli Alimenti di Origine Animale, University of Perugia, Perugia, 06126 Italy; 4 CIRAD, UMR ASTRE, Petit-Bourg, F-97170 France; 5 ASTRE, Université de Montpellier, CIRAD INRAE, Montpellier, France; 6 Gauteng Department of Agriculture and Rural Development, Johannesburg, 2001 South Africa

**Keywords:** Antimicrobials, Growth promoters, Induction, *Stx*-converting, Bacteriophages, STEC O157:H7

## Abstract

In this study, four antimicrobial growth promoters, including virginiamycin, josamycin, flavophospholipol, poly 2-propenal 2-propenoic acid and ultraviolet light, were tested for their capacity to induce *stx*-bacteriophages in 47 Shiga toxin-producing *E. coli* O157:H7 isolates. Induced bacteriophages were characterized for shiga toxin subtypes and structural genes by PCR, DNA restriction fragment length polymorphisms (RFLP) and morphological features by electron microscopy. Bacteriophages were induced from 72.3% (34/47) of the STEC O157:H7 isolates tested. Bacteriophage induction rates per induction method were as follows: ultraviolet light, 53.2% (25/47); poly 2-propenal 2-propenoic acid, 42.6% (20/47); virginiamycin, 34.0% (16/47); josamycin, 34.0% (16/47); and flavophospholipol, 29.8% (14/47). A total of 98 bacteriophages were isolated, but only 59 were digestible by *Nde*I, revealing 40 RFLP profiles which could be subdivided in 12 phylogenetic subgroups. Among the 98 bacteriophages, *stx2a, stx2c* and *stx2d* were present in 85.7%, 94.9% and 36.7% of bacteriophages, respectively. The *Q*, *P*, *CIII*, *N1*, *N2* and IS1203 genes were found in 96.9%, 82.7%, 69.4%, 40.8%, 60.2% and 73.5% of the samples, respectively. Electron microscopy revealed four main representative morphologies which included three bacteriophages which all had long tails but different head morphologies: long hexagonal head, oval/oblong head and oval/circular head, and one bacteriophage with an icosahedral/hexagonal head with a short thick contractile tail. This study demonstrated that virginiamycin, josamycin, flavophospholipol and poly 2-propenal 2-propenoic acid induce genetically and morphologically diverse free *stx*-converting bacteriophages from STEC O157:H7. The possibility that these antimicrobial growth promoters may induce bacteriophages in vivo in animals and human hosts is a public health concern. Policies aimed at minimizing or banning the use of antimicrobial growth promoters should be promoted and implemented in countries where these compounds are still in use in animal agriculture.

**Supplementary Information:**

The online version contains supplementary material available at 10.1186/s13099-023-00590-9.

## Introduction

Shiga toxin-producing *Escherichia coli* O157:H7 (STEC) causes foodborne disease in humans characterized by watery or bloody diarrhea. In 5–10% of patients, human STEC disease has been associated with complications including hemorrhagic colitis (HC) and hemolytic uremic syndrome (HUS). Ruminants, including cattle, sheep and goats are the main reservoirs of STEC [1]. Humans acquire STEC infections by ingesting STEC-contaminated meat and dairy products, water or vegetables [2–4]. STEC can be also transmitted from person-to-person and by contact with infected ruminants [5].

The major virulence factors of STEC are two antigenically distinct bacteriophages-encoded Shiga toxins: *stx1* and *stx2*, with various genetic variants including four *stx1* (*stx1a*, *stx1c*, *stx1d and stx1e*) and 15 *stx2* (*stx2a, stx2b, stx2c, stx2d, stx2e, stx2f, stx2g, stx2h, stx2i, stx2j, st2k, st2l, stx2m, stx2n and stx2o*) subtypes [3, 6, 7]. The genetic structure of shiga toxin-converting bacteriophages (also termed *stx*-converting bacteriophages or *stx*-phages) is similar to that of lambdoid bacteriophages, with immediate early, delayed and late phase transcribed genes [8]. Bacteriophage genomes are composed of structural genes which encode proteins responsible for capsid, tail, tail fibers and spike formation. In addition, a number of structural genes encode proteins that regulate virion replication, assembly and release, and shiga toxin expression. Important structural genes include *Q*, *N* and N2, which encode transcriptional antiterminator and late antiterminator proteins, respectively [9, 10]. Additional genes located upstream of the *Q* antiterminator include the *cI* repressor and *P*, which are responsible for phage immunity and DNA replication, respectively [11, 12].

Previously, it was shown that antimicrobial growth promoters which are supplemented to animal feed at subinhibitory concentrations induce lysogenic *stx*-converting bacteriophages in the STEC chromosome [13]. Bacteriophage induction occurs when antimicrobials damage DNA and activate the bacterial SOS response, which interferes with virion replication [14, 15]. At the molecular level, activation of the SOS system leads to derepression of the bacteriophage repressor (CI), which triggers transcription of genes involved in bacteriophage assembly, bacteriolysis and release of free virion particles from STEC [16, 17]. This phenomenon is considered the main driver of STEC emergence and evolution [8, 16]. Clinically, the use of antimicrobials for treatment of STEC disease in humans has been linked to induction of *stx*-converting bacteriophages with subsequent increase in Stx production and severe STEC disease in humans [18–22].

Although antimicrobial growth promoters have been banned in the European Union since 2006 [23], these compounds are still used as in-feed additives for livestock growth promotion in many countries around the world, including South Africa. In South Africa, 29% of antimicrobials that are approved for use in livestock growth promotion include compounds banned in the European Union, such as virginiamycin, josamycin, flavophospholipol and poly 2-propenal 2-propenoic acid [24]. Poly 2-propenal 2-propenoic acid cross-links and inactivates surface lipoproteins in the bacterial cell wall leading to bacterial death by lysis [25]. Both virginiamycin, a streptogramin, and josamycin, a second-generation macrolide, inhibit protein synthesis by binding to the 50 S subunit of the bacterial ribosome [26–28]. Flavophospholipol is a glycolipid antibiotic which blocks bacterial cell wall synthesis by suppressing peptidoglycan glycosyltransferases [29].

In this study, the capacity of virginiamycin, josamycin, flavophospholipol, poly 2-propenal 2-propenoic acid, and ultraviolet (UV) light to induce *stx*-converting bacteriophages from STEC O157:H7 was investigated. The four antimicrobial compounds have never been evaluated for their capacity to induce *stx*-converting bacteriophages. Bacteriophage induction assays were conducted in vitro on a collection of 47 STEC O157:H7 isolates. Released bacteriophages were further characterized for possession of structural and *stx*-encoding genes, morphology and restriction fragment length polymorphisms (RFLPs).

## Results

### STEC O157:H7 characteristics and bacteriophage induction rates

**Fig. 1:**
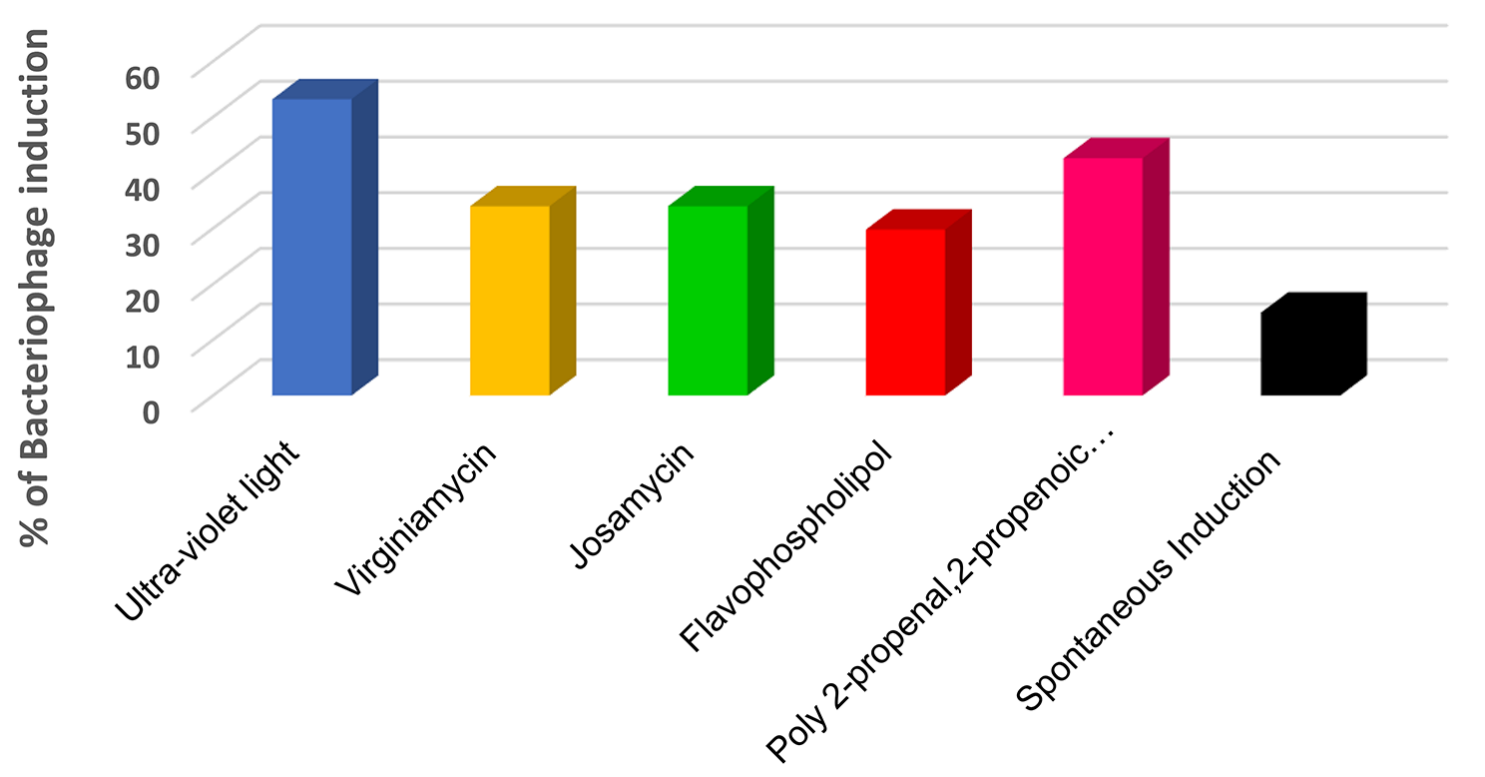
Stx-converting bacteriophages induction rates by UV and four antimicrobial growth promoters

Bacteriophages were induced from 34/47 (72.3%) STEC O157:H7 isolates tested. The rates of bacteriophage induced per induction method from the 47 STEC O157:H7 isolates were as follows (Fig. 1 and Table 1and **Supplementary Material Table****S1**): UV, 53.2% (25/47); poly 2-propenal 2-propenoic acid, 42.6% (20/47); josamycin, 34.0% (16/47); virginiamycin, 34.0% (16/47); and flavophospholipol, 29.8% (14/47). Only 14.9% (7/47) of STEC O157:H7 isolates tested released bacteriophages spontaneously.

**Fig. 2:**
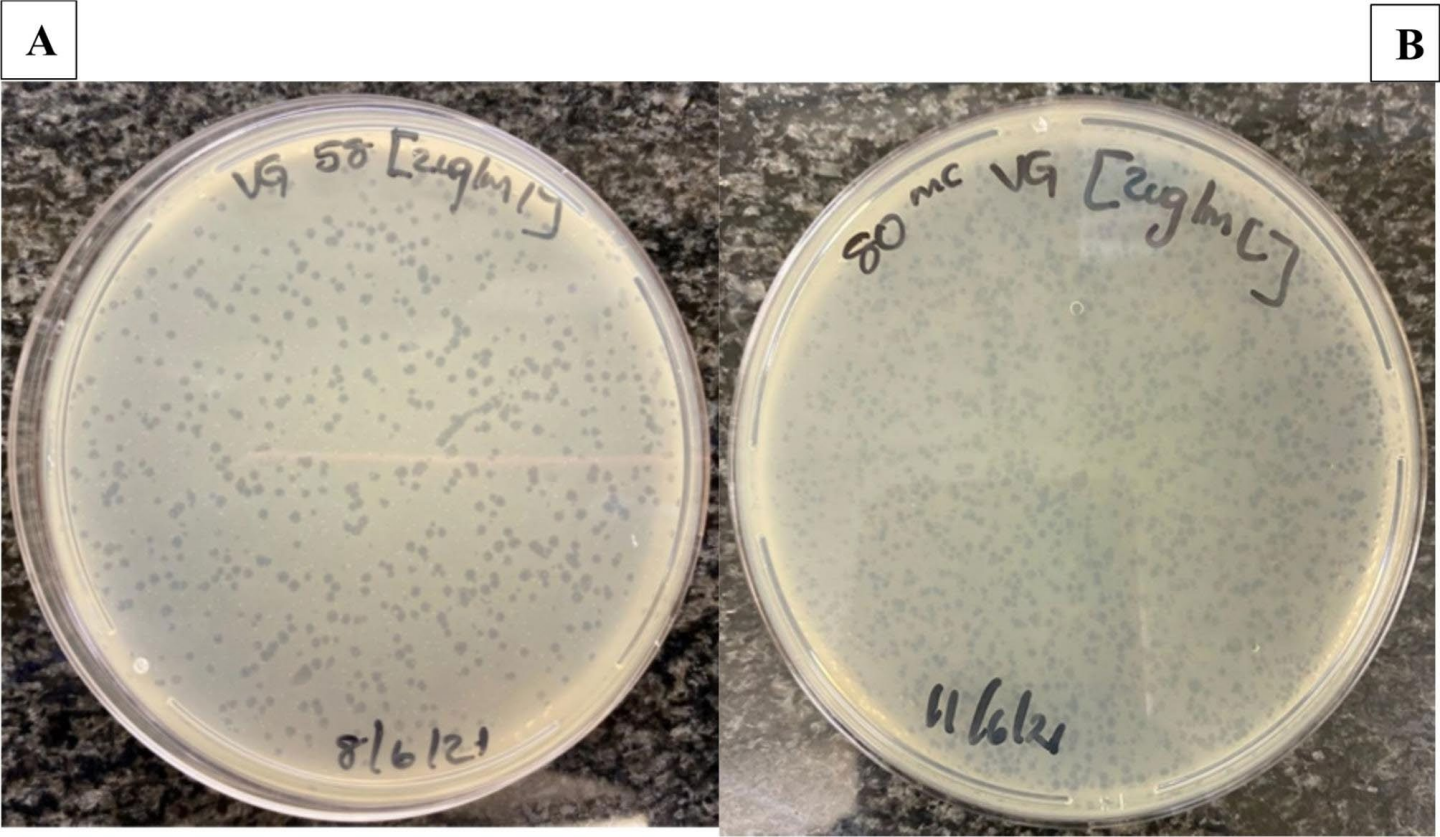
Bacteriophages plaques on agar plates after induction by virginiamycin

### Bacteriophage productivity scores per induction method

**Table 1:** Fig. 2. Bacteriophages productivity scores per induction method among the 47 STEC O157:H7 isolates

Bacteriophages infectivity scores	Ultraviolet light	Virginiamycin	Josamycin	Flavophospholipol	Poly 2-propenal 2-propenoic acid	Spontaneous
0 (0 plaques)	4 (16%)	5 (31,3%)	2 (12,5%)	1 (7,1%)	12 (60%)	0
1+ (< 5 plaques)	2 (8%)	4 (25%)	0	5 (35,7%)	1 (5%)	0
2+ (5 to 10 plaques)	15 (60%)	4 (25%)	10 (62,5%)	4 (28,6%)	2 (10%)	6 (85,7%)
3+ (> 10 plaques)	4 (16%)	3 (18,8%)	4 (25%)	4 (28,6%)	5 (25%)	1 (14,3%)
Total induced bacteriophages	**25/47**	**16/47**	**16/47**	**14/47**	**20/47**	**7/47**

### Distribution of bacteriophage-encoded genes

A total of 98 *stx*-converting bacteriophages were isolated and further genotyped for *stx*-encoding and structural genes (**Supplementary Material- Table****S1**). PCR genotyping revealed that all induced bacteriophages were *stx2* positive. Overall, the following rates of *stx-*encoding and structural genes were obtained from the 98 induced bacteriophages: *stx2*, 85,7%, (84/98); *stx2c*, 94,9% (93/98); and *stx2d*, 36.7%, (36/98). *P*, 96,9% (95/98); *Q*, 82.7%, (81/98); *CIII*, 69,4, (68/98); *N1*, 40,8 (40/98); *N2*, 60,2, (59/08); *IS1203/*Integrase, 73,5 (72/98) (**Supplementary Material- Table ****S1**).

**Fig. 3:**
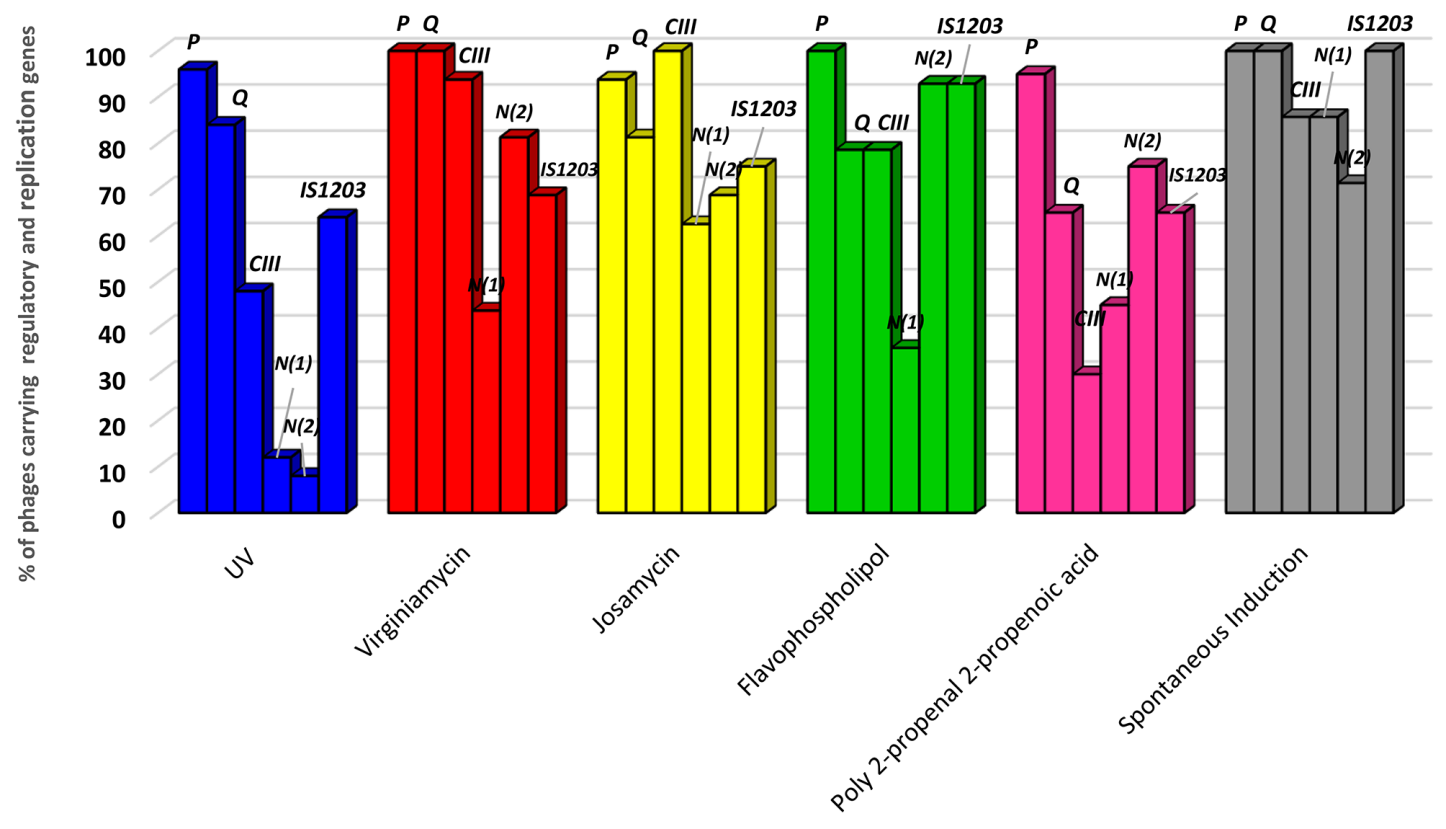
Distribution of structural genes encoded among on bacteriophages induced with different methods

**Fig. 4:**
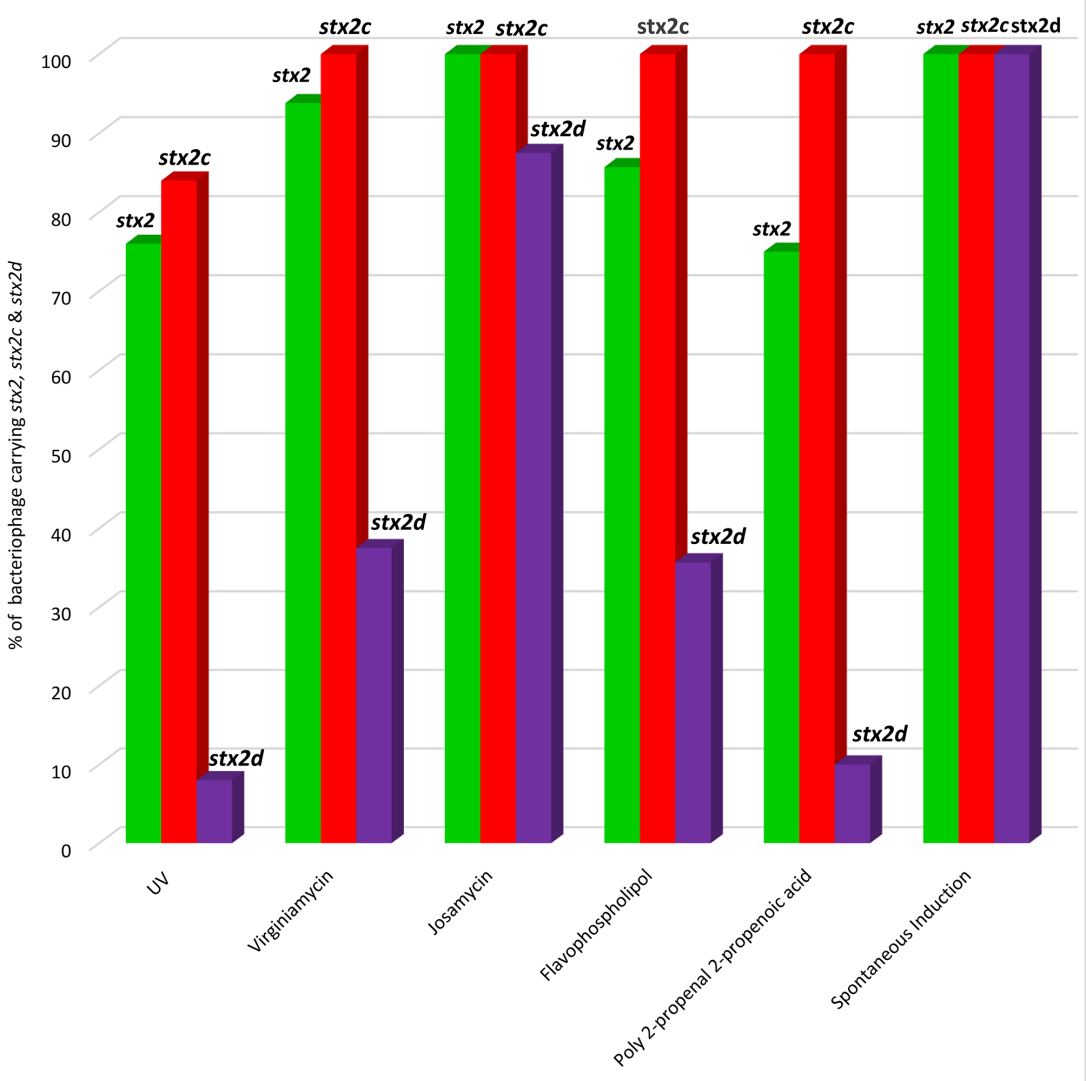
Distribution of *stx2* subtypes among induced bacteriophages

Bacteriophages productivity scores and presence of *stx*-encoding and structural genes in induced bacteriophages per induction method are depicted in Figs. 3 and 4; Table 1 and **Supplementary Materials Table****S1**. Among the 25 bacteriophages which were induced by UV, 76% (19/25) were *stx2* positive, 84% (21/25) carried *stx2c*, and 8% (2/25) carried *stx2d*. The following rates were obtained for genes coding for bacteriophage structural genes: *P*, 96% (24/25); *Q*, 84% (21/25); *CIII*, 48% (12/25); *N1*, 12% (3/25); *N2*, 8% (2/25); and *IS1203* (integrase), 64% (16/25).

Among the 20 bacteriophages induced by poly 2-propenal 2-propenoic, bacteriophage-encoded genes were observed at the following rates: *stx2*, 75% (15/20); *stx2c*, 100% (20/20), *stx2d*, 10% (2/20); *P*, 95% (19/20); *Q*, 65% (13/20); *CIII*, 30% (6/20); *N1*, 45% (9/20); *N2*, 75% (15/20); *IS1203*, 65% (13/20).

The 16 josamycin-induced bacteriophages revealed the following proportions of genes: *stx2*, 100% (16/16); *stx2c*, 100% (16/16), *stx2d*, 87.5% (14/16); *P*, 93.8% (15/16); *Q*, 81.3% (13/16); *CIII*, 100% (16/16); *N1*, 62.5% (10/16); *N2*, 68.8% (11/16); *IS1203* (integrase), 75% (12/16).

Virginiamycin-induced bacteriophages carried genes at the following rates: *stx2*, 93.8% (15/16); *stx2c*, 100% (16/16); *stx2d*, 37.5% (6/16); *P*, 100% (16/16); *Q*, 100% (16/16); *CIII*, 93.8% (15/16); *N1*, 43.8% (7/16); *N2*, 81.3% (13/16); and *IS1203* (integrase), 68.8% (11/16). The 14 bacteriophages that were induced by flavophospholipol had the following genes: *stx2*, 85.7% (12/14); *stx2c*, 100% (14/14); *stx2d*, 35.7% (5/14); *P*, 100% (14/14); *Q*, 78.6% (11/14); *CIII*, 92.9% (13/14); *N1*, 35.7% (5/14); *N2*, 92.9% (13/14); and *IS1203* (integrase), 92.9% (13/14). Among the 7 spontaneously induced bacteriophages, the following rates of genes were recorded: *stx2*, 100% (7/7); *stx2c*, 100% (7/7); *stx2d*, 100% (7/7); *P*, 100% (7/7); *Q*, 100% (7/7); *CIII*, 85.7% (6/7); *N1*, 85.7% (6/7); *N2*, 71.4% (5/7); and *IS1203* (integrase), 100% (7/7).

### Restriction fragment length polymorphism of bacteriophage DNA

**Fig. 5:**
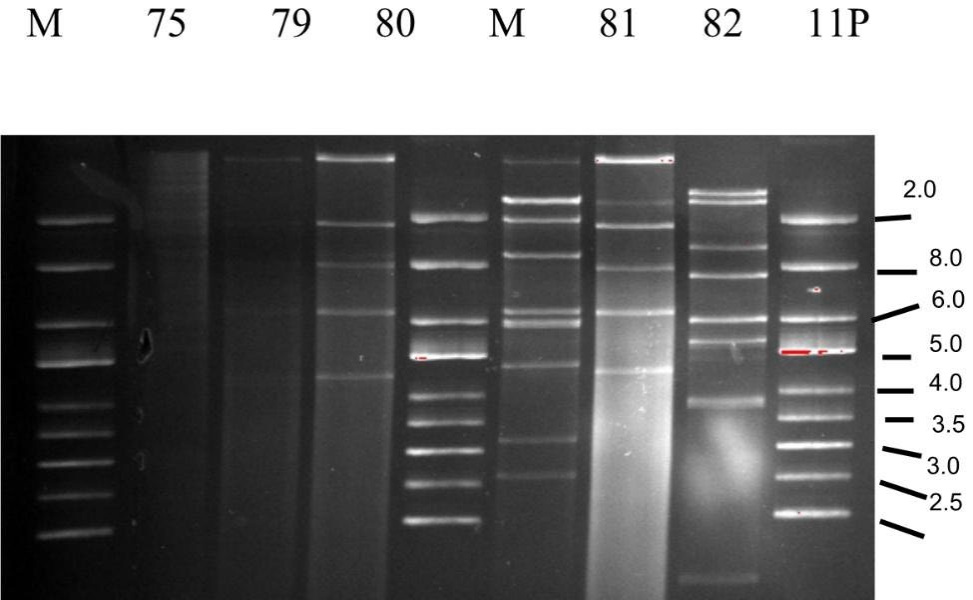
Electrophoresis in a 0.8% agarose gel digestion by NdeI restriction enzyme of Stx bacteriophage DNA induced by flavophospholipol and isolated from cattle O157:H7 strains. M - molecular size markers. The right side of the figure shows marker band sizes. Bacteriophages 75 and 79 showed very faint bands

**Fig. 6:**
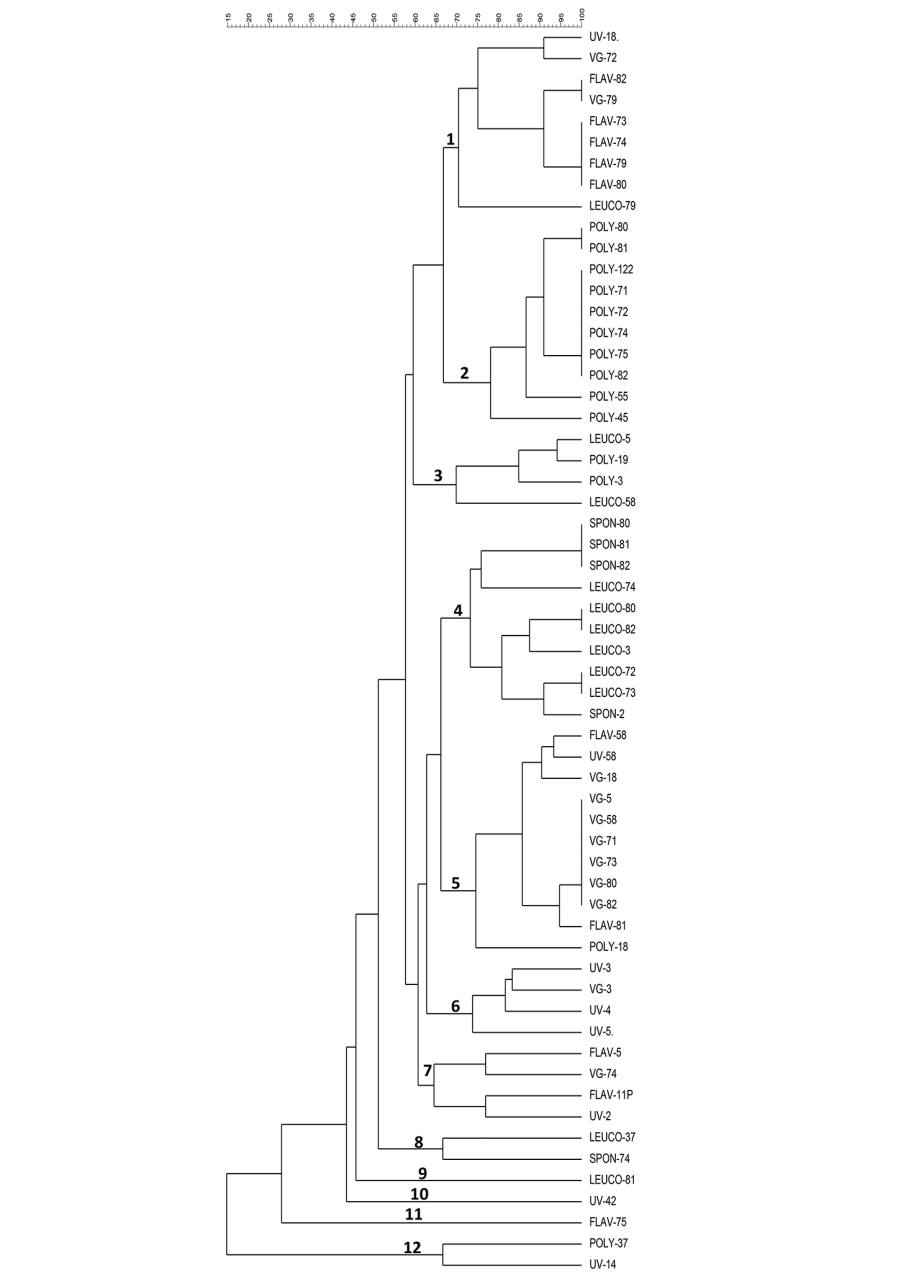
Dendogram depicting relatedness/diversity among bacteriophages generated from RFLP profiles by *NdeI* digestion

Bacteriophage DNA was digested using the restriction enzyme *Nde*I (Fig. 5), and a dendrogram was generated from RFLP digest gel images. Among the 98 induced bacteriophages, only 59 could be digested by *Nde*I. Analysis of the 59 RFLP profiles displayed 40 bacteriophage subtypes. The 40 subtypes could be assigned to 12 phylogenetic subgroups with a Dice similarity index ≥ 60% (Fig. 6). Among the 12 phylogenetic subgroups, three subgroups (9, 10 and 11) were each represented by one bacteriophage. Two subgroups (8 and 12) were represented by two bacteriophages each, three comprised 4 bacteriophages each (3, 6 and 7), one subgroup was represented by nine bacteriophages (subgroup 1), two subgroups comprised 10 bacteriophages each (subgroups 2 and 4) and one (subgroup 5) consisted of 11 bacteriophages.

### Morphological features and dimensions of bacteriophages

**Fig. 7:**
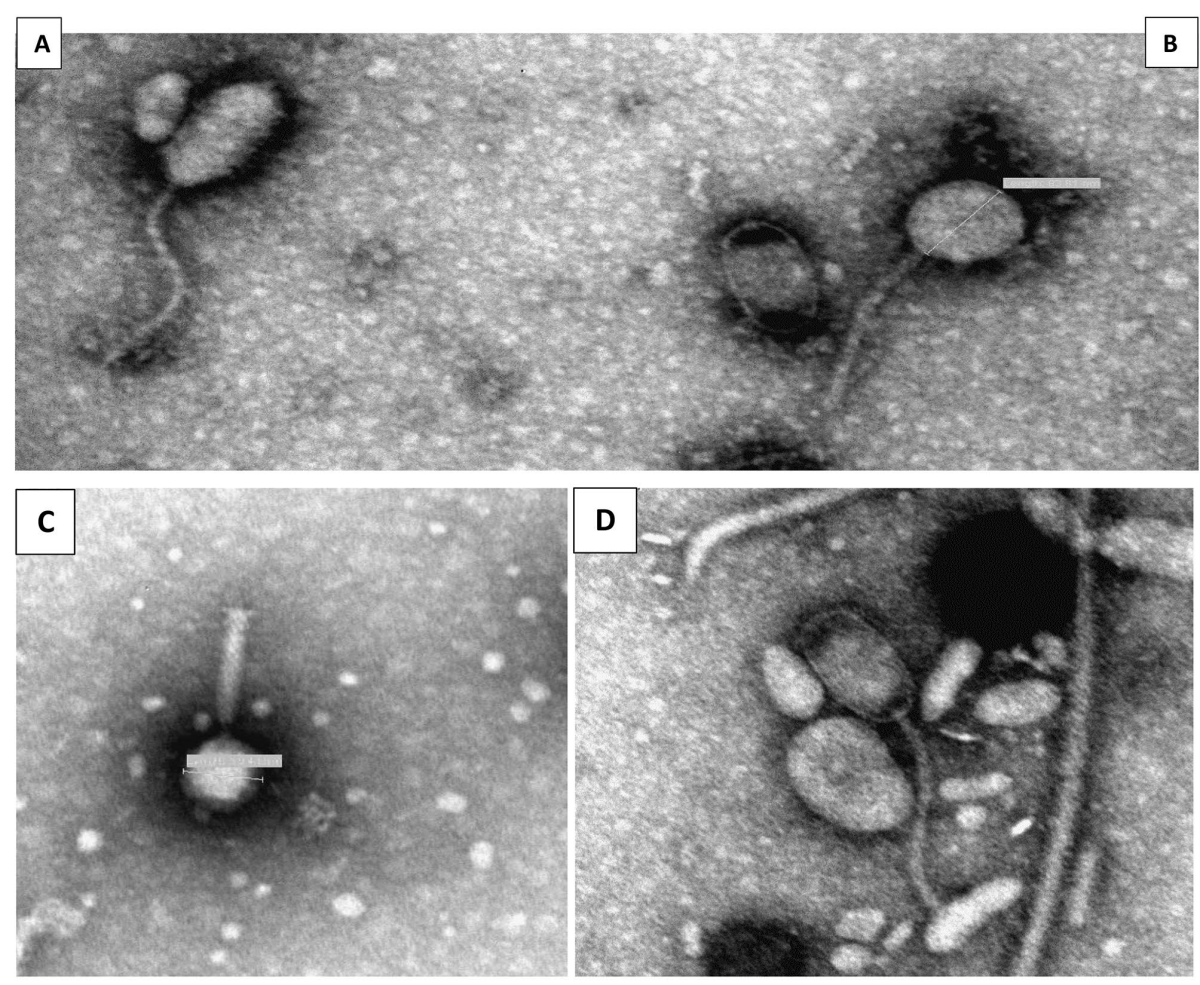
Electron micrographs of four bacteriophages. (**A**) Long hexagonal head with a long tail, (**B**) oval/circular head with a long tail. (**C**) Icosahedral/hexagonal head with a thick contractile tail. (**D**) Elongated (oblong) head with long tail. Bars = 200 nm

Electron microscopy revealed four morphologies: bacteriophages that possessed elongated icosahedral heads with long tails, oval heads with long tails and hexagonal heads with long tails and hexagonal heads with short thick contractile tail. All these bacteriophages lacked tail fibers (Fig. 7).

## Discussion

In this study, UV and four antimicrobial promoters, including poly 2-propenal 2-propenoic acid, josamycin, virginiamycin and flavophospholipol were tested for their capacity to induce bacteriophages in 47 STEC O157:H7 isolates from humans, cattle and goats. The four antimicrobials are approved for livestock growth promotion in South Africa but have never been tested for their capacity to induce bacteriophages.

Overall, it was possible to induce bacteriophages in 72.3% of the STEC O157:H7 isolates using UV light and the four antimicrobial promoters tested including poly 2-propenal 2-propenoic acid, josamycin, virginiamycin and flavophospholipol. As expected, the highest number of bacteriophages (53.2%) was induced by UV irradiation. UV light is considered a standard and effective inducer of *stx*-converting bacteriophages [30–33]. Zhang et al. [34]. Zhang et al. [34] compared bacteriophage induction by UV irradiation and a number of antimicrobials and showed that UV irradiation induced bacteriophages in the highest number of STEC isolates.

All the four antimicrobial growth promoters which were tested in this study induced plaque-forming *stx*-converting bacteriophages **(**Fig. 2**)**. However, bacteriophage induction rates and productivity scores were highly variable among induced STEC O157:H7 isolates. Poly 2-propenal 2-propenoic acid induced bacteriophages in the highest number of STEC O157:H7 (42.6%), followed by virginiamycin and josamycin (34,0%) and flavophospholipol (29.2%). This finding is consistent with other studies that have also observed variable bacteriophages induction capacity rates among antimicrobials [13, 19, 22, 35–37]. However, in contrast to previous studies that have shown that *stx*-converting bacteriophages are mainly induced by DNA-damaging antimicrobials that activate the SOS response [22, 35, 36, 38], this study demonstrated that bacteriophages could also be induced by protein (poly 2-propenal 2-propenoic acid, virginiamycin, josamycin) [25, 26, 28] and peptidoglycan synthesis (flavophospholipol) [29, 39] inhibiting antimicrobials. Previous studies have shown that antimicrobial agents that block bacterial cell wall or peptiglycan formation or protein synthesis either had no effect, suppressed or decreased *stx*-bacteriophage induction [35, 40–42]. While it remains unclear why there are differences in bacteriophages induction rates between this study and previous studies which used antimicrobials that inhibit protein and peptidoglycan synthesis capacity in bacterial hosts, variations in bacteriophages induction rates may have been influenced by the use of inadequate or suboptimal antimicrobial subinhibitory concentrations to induce bacteriophages in previous studies [26, 27, 29, 39]. Furthermore, the observed variations may be due to yet unexplained or unknown factors which are associated with intrinsic characteristics of the STEC O157:H7 isolates induced.

Similarly, as for bacteriophages induction rates, variations in bacteriophages productivity scores were also observed for different antimicrobials used to induce bacteriophages. The majority of isolates induced by josamycin and UV produced the highest number of bacteriophage plaques (more than 10 plaques/isolate). In contrast, the lowest bacteriophage productivity scores (≤ 5 plaques/isolate) were observed when poly 2-propenal 2-propenoic acid was used to induce bacteriophages, although this compound induced bacteriophages in the highest number of STEC O157:H7 isolates. According to Abedon and Culler, [43], during bacteriophage induction, high bacteriophages (plaque) productivity scores are a consequence of an optimal and long bacteriophage latent period, while low plaque productivity scores have been associated with a shorter bacteriophage latent period. Once again, while factors which influence variations in bacteriophage productivity scores cannot be explained, we suggest that bacteriophage productivity scores may also have been positively or negatively influenced by the use of nonptimal subinhibitory concentrations of antimicrobials used to induce bacteriophages.

It is difficult to compare our findings with other studies regarding the capacity of different antimicrobials to induce bacteriophages or influence virion productivity scores because previously similar bacteriophage induction studies were conducted on very small numbers of STEC isolates to allow valid comparisons with the our results [35, 40–42, 44]. Moreover, these investigations have yielded mixed results which cannot be compared with this study, depending on whether a direct or indirect method was used to measure *stx*-bacteriophage induction levels [35, 40–42, 44]. In addition, in this study, bacteriophage induction was assessed by the plaque assay technique, which is a direct method for measuring bacteriophage induction and productivity scores. This is unlike previous studies which demonstrated bacteriophage induction using indirect methods by measuring mRNA transcription levels, Stx expression levels or production of free Stx [19, 35, 45]. Differences in *stx*-bacteriophages induction rates and productivity scores among the STEC O157:H7 isolates tested may be a reflection of variations in STEC virulence capacity, toxin production levels and disease severity manifestations in human hosts, which can range from mild diarrhea to severe bloody diarrhea and complications such as HC and HUS.

Our results also showed that it was possible to induce *stx*-bacteriophages spontaneously in a small number of STEC O157:H7 isolates (14%), consistent with previous studies that have shown that *stx*-bacteriophages can be spontaneously induced from a small number of STEC O157:H7 strains [46, 47] under the influence of yet unknown environmental signals, sometimes independent of the RecA-dependent SOS response system [48]. Previous reports have shown that lysogens encoding *stx1* or *stx2* spontaneously released approximately 1 in 20 000–70 000 virion particles per cell generation [48, 49]. Bullwinkle et al., suggested that spontaneous induction occurs as a result of suboptimal concentrations of repressor needed to activate lytic functions in *stx*-bacteriophages [50, 51]. It appears that some *stx*-converting bacteriophages are evolutionarily selected for spontaneous induction in comparison to bacteriophages which require chemical or physical induction to be induced.

There were 27.7% of STEC O157:H7 isolates which did not induce any bacteriophages. STEC O157:H7 isolates that could not produce bacteriophages may have defective promoters that lack the switch from the lysogenic to the lytic state. Furthermore, bacteriophage induction failure and unsuccessful plaquing capacity may be ascribed to expression of colicins that are lethal to *E. coli* strains used for bacteriophage propagation [32]. In addition, some isolates may have not been able to produce bacteriophages because of an unsuitable, immune or insensitive bacteriophage propagation strain.

All 98 induced bacteriophages carried *stx2*, including 75% that possessed *stx2c* while a far lower number (36.7%) possessed *stx2d*. The high frequency of *stx2/stx2c-*encoding bacteriophages and a lower rate of *stx2d*-encoding virion particles was not surprising as these results corresponded to the original characteristics of STEC O157:H7 isolates induced in this study which have been reported elsewhere [52–54]. Higher induction rates of *stx2* followed by *stx2c*-encoding bacteriophages and a lower number of *stx2d*-positive virion particles is consistent with previous studies which observed that *stx2* and *stx2c*-encoding bacteriophages were more readily inducible in comparison to *stx2d*-carrying phages [47, 55, 56]. Furthermore, Fitzgerald et al., [57] reported that *stx2*-encoding bacteriophages were more frequently induced from STEC O157:H7 than *stx2c*. Also, previous reports on the molecular epidemiology of clinical STEC isolates have associated STEC strains that possess *stx2* and *stx2c* with a higher likelihood of severe disease occurrence in humans including HUS [58, 59]. Moreover, a finding of lower rates of *stx2d*-encoding bacteriophages was consistent with Gobius et al. [56] who observed that *stx2d*-encoding bacteriophages may be noninducible because they are carried on cryptic prophages remnants that lack genes responsible for activation from lysogeny to the lytic replicative cycle.

Out of the 98 induced bacteriophages, only 59 were digestible with the *Nde*I restriction enzyme, while the remaining 39 could not be cut by *NdeI*. Analysis of restriction fragment length polymorphism patterns showed that the 59 bacteriophage restriction profiles belonged to 12 major subgroups, reflecting the diversity (Dice similarity index ≥ 60%) among *stx*-converting STEC O157:H7 bacteriophages. Previous reports have also shown that *stx*-converting bacteriophages are very heterogeneous [12, 31, 60, 61]. Furthermore, a small portion of bacteriophages which were induced by poly 2-propenal 2-propenoic acid (cluster 2) and leucomycin (cluster 5) induction displayed identical (100%) restriction patterns. There was also a cluster of very closely related bacteriophages which were induced by flavophospholipol (cluster 1). Bacteriophages that clustered together or closely related were released from STEC O157:H7 isolates that originated from the same farm, suggesting the circulation of identical or closely related STEC O157:H7 strains and bacteriophages on farms where these isolates were collected.

Genotyping revealed that the majority of bacteriophages carried the *P* (attachment), *Q* (antiterminator), *CIII* (repressor) *N(1)* and *IS1234* (integrase) genes, while the *N(2)* gene, which also codes for integrase was amplified in only 40.8% of bacteriophages, independent of the bacteriophage induction method. This is consistent with previous studies which observed that structural genes are mostly conserved among *stx*-converting bacteriophages of STEC O157:H7 which usually share a common genetic regulatory system [9, 55, 60, 62, 63]. However, while structural genes of *stx-*converting bacteriophages are mostly conserved, variations among these genes have been observed depending on the origin of the STEC O157:H7 isolates harboring them, as previously shown in studies which compared fly with cattle isolates [62] and clinical versus bovine STEC strains [63]. The presence of structural genes among *stx*-bacteriophages may also vary depending on the subtype of the gene (Q21 vs. *Q33*) [63] or whether a particular gene subtype is truncated or complete [64]. Furthermore, Llarena et al., (2021) [65] reported new non-lambdoid *stx*-converting bacteriophages which carried yet undescribed novel sequences of *P* replication initiation genes.

Electron microscopy revealed four main groups of *stx*-converting bacteriophages morphologies including three which all had a long tail with the following representative head shapes: a long hexagonal head, oval/circular head, and elongated (oblong/prolate) head [31, 66, 67]. A fourth group of bacteriophages had an icosahedral/hexagonal head with a short thick contractile tail. The morphological features of bacteriophages which were observed in this study agreed with previous studies, which found that most *stx*-converting bacteriophages either had elongated or oval heads with long tails or regular hexagonal heads with short tails [31, 66, 67].

To our knowledge, this is the first study reporting on the capacity of virginiamycin, josamycin, flavophospholipol and poly 2-propenal 2-propenoic acid to induce bacteriophages from STEC O157:H7 isolates. Our results demonstrated that these four antimicrobials induce *stx*-converting bacteriophages, which are genetically and morphologically diverse. The induced *stx*-bacteriophages encoded *stx2* and *stx2c* mostly and *stx2d* to a lesser extent. The use of these antimicrobial promoters as in-feed additives in South Africa and other countries around the world may be contributing to STEC emergence and expansion and evolution of new STEC by converting naïve *E. coli* into STEC through lateral gene transfer. This is a public health concern that warrants the formulation of evidence-based policies aimed at stimulating the prudent use of antimicrobials in livestock husbandry. Finding alternative antimicrobial promoters that do not compromise public health or an altogether ban of these compounds in South Africa and other countries where they are still used in animal Agriculture is recommended.

## Materials and methods

### Bacterial strains

A total of 47 STEC O157:H7 isolates were used for the induction of *stx*-converting bacteriophages. The STEC O157:H7 isolates included 34 cattle (25 beef + 9 dairy), six human and seven goat isolates. Before conducting bacteriophage induction studies, the 47 isolates were reconfirmed as STEC O157:H7 [68, 69] and screened for *stx1*, *stx2, stx2c* and *stx2d* by PCR [70, 71]. Briefly, multiplex polymerase chain reaction (mPCR) was performed to detect *stx1* and *stx2* in the STEC isolates using previously described primers and cycling conditions [70]. The virulence characteristics of the STEC isolates used for bacteriophages induction were reported in previous studies [52–54].

### Bacteriophage induction with UV light

UV light was used as the reference standard bacteriophage induction method against which bacteriophage induction by antimicrobial growth promoters was compared. Bacteriophage induction with UV light irradiation was carried out according to previously published protocols [31]. Briefly, before bacteriophage induction assays, frozen STEC O157:H7 cultures were streaked on Luria Bertani (LB) agar (10 g tryptone, 5 g yeast extract, 10 g/L NaCl) and incubated at 37 °C overnight to obtain pure single colonies of STEC O157:H7. A single colony of STEC O157:H7 was added to a 250 ml Erlenmeyer baffled base culture flask (BD Biosciences, Erembodegem, Belgium) containing 45 ml of modified LB broth (10 g tryptone, 5 g yeast extract, 2.5 g/L NaCl and 0.01 M CaCl_2_). The broth was incubated at 37 °C with shaking at 200 rpm for 4 h to attain exponential growth. After four hours of incubation, the bacterial culture was centrifuged at 4000 rpm for 45 min. The pellet was suspended in 5 ml of 0.01 M CaCl_2_ and transferred onto a glass petri dish. Thereafter, bacteriophage induction was carried out by UV irradiation of the STEC O157:H7 strains according to a previously described protocol [31]. Briefly, the petri dish containing the bacterial suspension was placed inside a biosafety cabinet (Esco AC2-4S1, South Africa) under a UV lamp at 40 cm (Esco, UV-30 A, South Africa). The front movable window was covered with aluminum foil to create a dark chamber. Bacteriophage induction was performed by irradiating the bacterial suspension for 120 s with UV light. The UV light wavelength was 254 nm. The irradiated bacterial suspension was transferred into a sterile 250 mL Erlenmeyer baffled base culture flask (BD Biosciences, Erembodegem, Belgium) containing 45 mL of modified LB broth and incubated at 37 °C with shaking (200 rpm) overnight. The overnight culture was centrifuged at 4000 rpm for 45 min, and the supernatant was filtered in a 50 ml centrifuge tube through a 0.45-µm pore-size membrane. Two to three drops of chloroform were added to the filtrate. The filtrate was stored at 4 °C until further processing.

### Bacteriophage induction with antimicrobial growth promoters

Four antimicrobial growth promoters were tested for their capacity to induce bacteriophages, including josamycin (leucomycin), virginiamycin, flavophospholipol and poly 2-propenol 2-propenoic acid (acrolein). All antimicrobials were supplied by Merck (Sigma‒Aldrich), South Africa, except for flavophospholipol, which was kindly donated by V-Tech Pty (Ltd), South Africa. Before bacteriophage induction, subinhibitory concentrations (SICs) of virginiamycin, josamycin (leucomycin A3), flavophospholipol and poly 2-propenal, 2-propenoic acid (acrolein) were determined based on previously published *E. coli* minimum inhibitory concentrations (MICs) [25, 72–74]. Bacteriophage induction using antimicrobials was similar to U.V. induction, with slight modifications. Briefly, STEC O157:H7 isolates were cultured in 45 ml of modified LB broth for 4 h with shaking at 200 rpm at 37 °C to attain the exponential growth phase. After 4 h, the exponential growing culture was centrifuged at 4000 X g for 5 min, the supernatant was discarded, and the bacterial pellet was suspended in 5 ml of a 0.01 CaCl_2_ solution. Bacteriophage induction was performed by adding SICs of antimicrobials to the 5 ml bacterial suspension: virginiamycin (2 µg/mL) [72], josamycin **(**128 µg/mL) [73] (flavophospholipol (64 µg/mL) [74] and poly 2-propenal 2-propenoic acid (3 µg/mL) [25]. The suspension was added to 45 ml of modified LB and incubated for 16-24 h with shaking at 200 rpm at 37 °C. After incubation, induced cultures were centrifuged at 4000 X g for 45 min, and the supernatant was filtered through a 0.45-µm pore-size membrane. Two to three drops of chloroform were added to the filtrate, which was stored at 4 °C until further processing. Spontaneous bacteriophage induction was also tested by culturing bacteria in modified LB broth for 20 h at 37 °C with shaking at 200 rpm followed by centrifugation of the broth at 4000 X g for 45 min followed by filtration of the supernatant through a 0.45-µm pore-size membrane. Two to three drops of chloroform were added to the filtrate which was stored at 4 °C, until further processing.

**Bacteriophage propagation**.

The double-layer agarose plaque assay technique for isolation and enumeration of phage ʎ was used to propagate bacteriophages and isolate plaques [75]. Briefly, 100 µL of the supernatant of the induced culture filtrate was mixed with 100 µL of 0.01 M CaCl_2_ and 100 µL of an overnight culture of the *E. col*i K-12 MC1061 bacteriophage propagation strain. The mixture was incubated at 37 °C for 30 min to allow bacteriophage adsorption, gently mixed with 3 mL of soft agarose (100 mL of modified LB broth, 0.5 g agarose) and poured onto a petri dish containing 1.5% modified hard LB agarose (Modified LB, 1.2 g MgSO_4_.7H_2_O, 15 g agarose in 1 L of H_2_O). The overlay soft agarose was allowed to solidify and incubated at 37 °C overnight while being monitored for plaque formation and productivity for up to 48 h. To rank the different levels of plaque productivity, a scoring system based on the number of plaque-forming units (PFUs) on agarose plates was applied as follows: +3 > 10 plaques; +2 = 5 to 10 plaques; +1 < 5 plaques; and + 0 no plaques .

### Bacteriophage isolation

Single plaques were harvested from each plate showing plaques by aspirating a single plaque from the surface of the double layer agarose using a sterile glass Pasteur pipette. Aspirated plaques were individually suspended in Eppendorf tubes containing 500 µL of 0.01 M CaCl_2_ solution. A 100 µL aliquot of the plaque suspension was mixed with 100 µL of 0.01 M CaCl_2_ and 100 µL of the overnight culture of *E. col*i K-12 strain MC1061 and incubated for 30 min. The solution was gently mixed with 3 mL of soft agar, poured onto hard agar for bacteriophage propagation and incubated overnight at 37 °C. Bacteriophages were collected by pouring 5 mL of SM buffer solution (5.8 g NaCl, 2 g MgSO_4_.7H_2_O, 50 mL of 1 M Tris-Cl [pH 7.5], 5 mL of 2% gelatin, 1 L of H_2_O) onto petri dishes showing plaques. To obtain an adequate bacteriophage titer for DNA extraction, one plaque was multiplied on five petri dishes. Bacteriophage collection was carried out by pouring SM buffer (100 mM sodium chloride, 10 mM magnesium sulfate, 50 mM Tris-HCl, pH 7.5 and 0.01% (w/v) gelatin) on petri dishes showing plaques. To dislodge bacteriophages from the soft agar, the petri dishes were placed on a platform shaker (FMH Electronics, South Africa) and then incubated with soft shaking for 24–48 h at 4 °C. Bacteriophages were harvested by scratching off the top soft agarose from the hard agar and transferring the soft agar/SM buffer suspension mixture to a 50 mL centrifuge tube. Soft agar/SM buffer suspensions from a common single plaque were pooled in a 50 mL tube to obtain an adequate phage titer and centrifuged at 4000 X g for 30 min. The supernatant was filtered using a 0.45-µm pore-size membrane and transferred into a sterile 50 mL tube. To eliminate any probable residual bacterial contamination, two to three drops of chloroform were added to the bacteriophage filtrate, which was stored at 4 °C.

### Bacteriophage DNA Extraction

DNA was extracted from bacteriophage lysate filtrates using the Qiagen Plasmid Midi Kit (Qiagen, Hilden, Germany) supplementary protocol for isolation of single-stranded DNA from M13 phage, according to the manufacturer’s instructions. The protocol is based on inactivation of bacterial DNA and RNA in a bacteriophage lysate with DNase and RNase, respectively, followed by precipitation of bacteriophage particles with 30% polyethylene glycol 8000 (Merck, South Africa), lysis of bacteriophages to release DNA, several DNA washings, DNA binding to an anion-exchange resin, DNA elution and precipitation and solution of the precipitated DNA in TE buffer (10 mM Tris, 1 mM EDTA, pH 8).

### Genetic characterization of induced bacteriophages from STEC O157:H7

PCR was used to screen bacteriophage DNA for genes encoding shiga toxins (*stx1*, *stx1c*, *stx1d*, *stx2*, *stx2c* and *stx2d*) and bacteriophage structural genes (*P, Q, CIII, N1, N2* and *IS1203*/integrase) [17, 60, 76]. In addition, to ensure that bacteriophage DNA was not contaminated with STEC bacterial chromosomal DNA, bacteriophage DNA was screened for the chromosomally encoded *eaeA* and *hlyA* genes [70]. The PCRs consisted of 25 µL containing 2.5 µL of 10X Thermopol reaction buffer, 2.0 µL of 2.5 mM dNTPs (deoxynucleotide triphosphates), 0.25 µL of 100 mM MgCl_2_, 0.6 µL of each primer (10 µM final concentration), 1 U of Taq DNA Polymerase and 5 µL of DNA template. All PCR reagents were procured from New England BioLabs (NEB, Ipswich, MA, USA) except for the primers, which were supplied by Inqaba Biotec (Pretoria, South Africa). DNA from the EDL933 (*E. coli* 0157:H7) strain and sterile **molecular grade** water were used as positive and negative controls, respectively, in all PCRs.

### Restriction fragment length polymorphism profiling

RFLP profiling (RFLP) of bacteriophage DNA was carried out by digesting 5 µL of bacteriophage DNA with the *NdeI* restriction enzyme (New England BioLabs, Ipswich, MA, USA) according to the manufacturer’s instructions. Bacteriophage DNA was separated by electrophoresis in a 0.8% agarose gel, stained with ethidium bromide (0.5 µg/ml) and visualized with UV light in a Gel Doc system (Bio-Rad, USA). Bionumerics software (Applied Maths, Sint Martens-Latem, Belgium) was used to analyze RFLP patterns and construct dendrograms based on the Dice similarity index (complete linkage, optimization, 1.5%; position tolerance 1.5) and the unweighted pair group method with arithmetic mean (UPGMA).

### Determination of bacteriophage morphology by electron microscopy

For morphological characterization of bacteriophages, bacteriophages were negatively stained with 3% phosphotungstic acid and examined by electron microscopy (EM). Briefly, a 1 mL suspension of phage supernatant was centrifuged in a Sigma 1–16 ultracentrifuge for 45 min, and the pellet was resuspended in **sterile molecular grade** water and a drop of the suspension deposited on a 300-mesh formvar-coated copper grid. The grid was negatively stained with 3% phosphotungstic acid and examined at 80 kV with a Philips CM10 transmission electron microscope at the EM Unit, Department of Anatomy and Physiology, Faculty of Veterinary Science, University of Pretoria.

### Electronic supplementary material

Below is the link to the electronic supplementary material.

Supplementary Material 1: Table S1 Characteristics of *stx*-converting bacteriophages induced from STEC O157:H7 isolates.
