# Supplementary material for: Antimicrobial growth promoters approved in food-producing animals in South Africa induce shiga toxin-converting bacteriophages from Escherichia coli O157:H7
Source: Gut Pathog. 2023 Dec 6;15:64. doi: 10.1186/s13099-023-00590-9 (PMC10698906; doi:10.1186/s13099-023-00590-9)
Supplement: Supplementary file 1 — Supplementary Material 1: Table S1 Characteristics of stx-converting bacteriophages induced from STEC O157:H7 isolates. [file 13099_2023_590_MOESM1_ESM.docx]

**Supplementary Material. Table S1.** Characteristics of *stx*-converting bacteriophages induced from STEC O157:H7 isolates.

| **Phages Induced** | **Farm** | **Origin** | ***Nde*I** | **Bacteriophage characteristics** | | | | | | | | |
| --- | --- | --- | --- | --- | --- | --- | --- | --- | --- | --- | --- | --- |
|  |  |  |  | ***stx2*** | ***stx2c*** | ***stx2d*** | ***P*** | ***Q*** | ***CIII*** | ***N(1)*** | ***N(2)*** | ***IS1203*** |
| UV-1 | A | Human | - | + | + | - | + | - | - | - | - | + |
| UV-2 | A | Human | + | + | + | - | + | + | + | - | - | + |
| UV-3 | A | Human | + | + | + | - | + | + | + | - | + | + |
| UV-4 | A | Human | + | + | + | - | + | + | + | - | + | + |
| UV-5 | A | Human | + | + | + | - | + | + | + | - | - | + |
| UV-6 | B | Cattle | - | + | + | - | + | + | + | - | - | + |
| UV-7 | B | Cattle | - | + | + | - | + | + | + | - | - | + |
| UV-8 | C | Cattle | - | - | - | - | + | + | - | - | - | + |
| UV-9 | C | Cattle | - | + | + | - | + | + | - | - | - | + |
| UV-10 | D | Cattle | - | + | + | - | + | + | - | - | - | + |
| UV-11 | C | Cattle | - | - | + | - | + | - | - | - | - | + |
| UV-12 | C | Cattle | - | - | + | - | + | - | - | + | - | + |
| UV-13 | C | Cattle | - | - | + | - | + | + | - | - | - | - |
| UV-14 | C | Cattle | + | + | + | - | + | + | + | - | - | + |
| UV-16 | C | Cattle | - | + | + | - | + | + | - | - | - | - |
| UV-17 | C | Cattle | - | - | + | - | + | + | - | - | - | - |
| UV-18 | C | Cattle | + | + | - | - | + | + | + | - | - | - |
| UV-19 | B | Cattle | - | + | - | - | + | + | - | - | - | - |
| UV-37 | A | Human | - | + | - | - | - | - | - | - | - | - |
| UV-42 | D | Cattle | + | + | + | - | + | + | - | - | - | - |
| UV-58 | C | Cattle | + | - | + | - | + | + | + | - | - | - |
| UV-72 | E | Cattle | - | + | + | + | + | + | + | + | - | + |
| UV-75 | E | Cattle | - | + | - | - | + | + | + | - | - | - |
| UV-81 | E | Cattle | - | + | + | + | + | + | + | + | - | + |
| UV-124 | C | Goat | - | + | + | - | + | + | - | - | - | + |
| **Subtotal (%)** |  |  | **8/25 (32)** | **19/25 (76)** | **20/25 (80)** | **2/25 (8)** | **24/25 (96)** | **21/25 (84)** | **12/25 (48)** | **3/25 (12)** | **2/25 (8)** | **16/25 (64)** |
|  |  |  |  | ***stx2*** | ***stx2c*** | ***stx2d*** | ***P*** | ***Q*** | ***CIII*** | ***N(1)*** | ***N(2)*** | ***IS1203*** |
| VG-3 | A | Human | + | + | + | + | + | + | + | - | + | - |
| VG-5 | A | Human | + | + | + | - | + | + | + | - | + | + |
| VG-11 | C | Cattle | - | + | + | - | + | + | - | - | - | - |
| VG-14 | C | Cattle | - | + | + | - | + | + | + | - | + | + |
| VG-18 | C | Cattle | + | - | + | - | + | + | + | - | + | - |
| VG-37 | A | Human | - | + | + | - | + | + | + | - | - | - |
| VG-58 | C | Cattle | + | + | + | - | + | + | + | - | + | + |
| VG-71 | E | Cattle | + | + | + | + | + | + | + | - | + | + |
| VG-72 | E | Cattle | + | + | + | + | + | + | + | + | + | + |
| VG-73 | E | Cattle | + | + | + | + | + | + | + | + | + | + |
| VG-74 | E | Cattle | + | + | + | - | + | + | + | + | + | + |
| VG-75 | E | Cattle | - | + | + | - | + | + | + | - | + | - |
| VG-79 | E | Cattle | + | + | + | - | + | + | + | + | + | + |
| VG-80 | E | Cattle | + | + | + | - | + | + | + | + | + | + |
| VG-81 | E | Cattle | - | + | + | + | + | + | + | + | - | + |
| VG-82 | E | Cattle | + | + | + | + | + | + | + | + | + | + |
| **Subtotal (%)** |  |  | **11/16 (68,8)** | **15/16 (93,8)** | **16/16 (100)** | **6/16 (37,5)** | **16/16 (100)** | **16/16 (100)** | **15/16 (93,8)** | **7/16 (43,8)** | **13/16 (81,3)** | **11/16 (68,8)** |
|  |  |  |  | ***stx2*** | ***stx2c*** | ***stx2d*** | ***P*** | ***Q*** | ***CIII*** | ***N(1)*** | ***N(2)*** | ***IS1203*** |
| LEUCO-3 | A | Human | + | + | + | + | + | + | + | + | + | + |
| LEUCO-5 | A | Human | + | + | + | + | + | + | + | - | + | + |
| LEUCO-11 | C | Cattle | - | + | + | - | - | - | + | - | - | - |
| LEUCO-12 | C | Cattle | - | + | + | + | + | - | + | - | - | + |
| LEUCO-14 | C | Cattle | - | + | + | + | + | - | + | - | - | + |
| LEUCO-18 | C | Cattle | - | + | + | + | + | + | + | - | + | - |
| LEUCO-37 | A | Human | + | + | + | + | + | + | + | - | - | - |
| LEUCO-58 | C | Cattle | + | + | + | + | + | + | + | + | + | - |
| LEUCO-72 | E | Cattle | + | + | + | + | + | + | + | + | + | + |
| LEUCO-73 | E | Cattle | + | + | + | + | + | + | + | + | + | + |
| LEUCO-74 | E | Cattle | + | + | + | + | + | + | + | + | + | + |
| LEUCO-75 | E | Cattle | - | + | + | + | + | + | + | + | - | + |
| LEUCO-79 | E | Cattle | + | + | + | + | + | + | + | + | + | + |
| LEUCO-80 | E | Cattle | + | + | + | - | + | + | + | + | + | + |
| LEUCO-81 | E | Cattle | + | + | + | + | + | + | + | + | + | + |
| LEUCO-82 | E | Cattle | + | + | + | + | + | + | + | + | + | + |
| **Subtotal (%)** |  |  | **11/16 (68,8)** | **16/16 (100)** | **16/16 (100)** | **14/16 (87,5)** | **15/16 (93,8)** | **13/16 (81,3)** | **16/16 (100)** | **10/16 (62,5)** | **11/16 (68,8)** | **12/16 (75)** |
|  |  |  |  | ***stx2*** | ***stx2c*** | ***stx2d*** | ***P*** | ***Q*** | ***CIII*** | ***N(1)*** | ***N(2)*** | ***IS1203*** |
| FLAV-3 | A | Human | - | + | + | + | + | - | + | - | + | + |
| FLAV-5 | A | Human | + | + | + | + | + | + | + | - | + | + |
| FLAV-11P | C | Cattle | + | + | + | - | + | + | - | - | + | + |
| FLAV-12 | C | Cattle | - | + | + | - | + | - | + | - | + | + |
| FLAV-14 | C | Cattle | - | - | + | - | + | - | + | - | + | - |
| FLAV-58 | C | Cattle | + | - | + | - | + | + | + | - | + | + |
| FLAV-72 | E | Cattle | - | + | + | - | + | + | + | - | + | + |
| FLAV-73 | E | Cattle | + | + | + | + | + | + | + | + | + | + |
| FLAV-74 | E | Cattle | + | + | + | - | + | + | + | + | - | + |
| FLAV-75 | E | Cattle | + | + | + | - | + | + | + | - | + | + |
| FLAV-79 | E | Cattle | + | + | + | + | + | + | + | - | + | + |
| FLAV-80 | E | Cattle | + | + | + | - | + | + | + | + | + | + |
| FLAV-81 | E | Cattle | + | + | + | - | + | + | + | + | + | + |
| FLAV-82 | E | Cattle | + | + | + | + | + | + | + | + | + | + |
| **Subtotal (%)** |  |  | **10/14 (71,4)** | **12/14 (85,7)** | **14/14 (100)** | **5/14 (35,7)** | **14/14 (100)** | **11/14 (78,6)** | **13/14 (92,9)** | **5/14 (35,7)** | **13/14 (92,9)** | **13/14 (92,9)** |
|  |  |  |  | ***stx2*** | ***stx2c*** | ***stx2d*** | ***P*** | ***Q*** | ***CIII*** | ***N(1)*** | ***N(2)*** | ***IS1203*** |
| POLY-3 | A | Human | + | + | + | + | + | + | + | + | + | + |
| POLY-11 | C | Cattle | - | - | + | - | + | - | - | - | - | - |
| POLY-12 | C | Cattle | - | - | + | - | + | - | - | - | - | - |
| POLY-14 | C | Cattle | - | - | + | - | + | - | - | - | - | - |
| POLY-16 | C | Cattle | - | + | + | - | + | + | - | - | + | + |
| POLY-18 | C | Cattle | + | + | + | - | + | + | + | - | + | - |
| POLY-19 | B | Cattle | + | + | + | - | + | + | + | - | + | + |
| POLY-37 | A | Human | + | + | + | - | + | + | + | - | - | + |
| POLY-45 | D | Cattle | + | + | + | + | + | - | - | + | + | + |
| POLY-55 | F | Cattle | + | + | + | - | + | + | + | - | + | + |
| POLY-71 | E | Cattle | + | + | + | - | + | - | - | + | + | + |
| POLY-72 | E | Cattle | + | + | + | - | + | - | - | - | - | - |
| POLY-73 | E | Cattle | - | + | + | - | + | - | - | - | + | - |
| POLY-74 | E | Cattle | + | + | + | - | + | + | + | - | + | + |
| POLY-75 | E | Cattle | + | + | + | - | + | + | - | + | + | + |
| POLY-79 | E | Cattle | - | + | + | - | + | + | - | + | + | + |
| POLY-80 | E | Cattle | + | + | + | - | + | + | - | + | + | + |
| POLY-81 | E | Cattle | + | + | + | - | + | + | - | + | + | + |
| POLY-82 | E | Cattle | + | - | + | - | - | + | - | + | + | + |
| POLY-122 | C | Goat | + | - | + | - | + | + | - | + | + | - |
| **Subtotal (%)** |  |  | **14/20 (70)** | **15/20 (75)** | **20/20 (100)** | **2/20 (10)** | **19/20 (95)** | **13/20 (65)** | **6/20 (30)** | **9/20 (45)** | **15/20 (75)** | **13/20 (65)** |
|  |  |  |  | ***stx2*** | ***stx2c*** | ***stx2d*** | ***P*** | ***Q*** | ***CIII*** | ***N(1)*** | ***N(2)*** | ***IS1203*** |
| SPON-2 | A | Human | + | + | + | + | + | + | - | - | + | + |
| SPON-37 | A | Human | - | + | + | + | + | + | + | + | - | + |
| SPON-74 | E | Cattle | + | + | + | + | + | + | + | + | + | + |
| SPON-75 | E | Cattle | - | + | + | + | + | + | + | + | - | + |
| SPON-80 | E | Cattle | + | + | + | + | + | + | + | + | + | + |
| SPON-81 | E | Cattle | + | + | + | + | + | + | + | + | + | + |
| SPON-82 | E | Cattle | + | + | + | + | + | + | + | + | + | + |
| **Subtotal (%)** |  |  | **5/7 (71,4)** | **7/7 (100)** | **7/7 (100)** | **7/7 (100)** | **7/7 (100)** | **7/7 (100)** | **6/7 (85,7)** | **6/7 (85,7)** | **5/7 (71,4)** | **7/7 (100)** |
| TOTAL of POSITIVES |  |  | **59/98** | **84/98** | **93/98** | **36/98** | **95/98** | **81/98** | **68/98** | **40/98** | **59/98** | **72/98** |
| **%** |  |  | **60,2** | **85,7** | **94,9** | **36,7** | **96,9** | **82,7** | **69,4** | **40,8** | **60,2** | **73,5** |
|  |  |  | ***Nde*I** | ***stx2*** | ***stx2c*** | ***stx2d*** | ***P*** | ***Q*** | ***CIII*** | ***N(1)*** | ***N(2)*** | ***IS1203*** |
